# Intrapleural Injection of Anti-PD1 Antibody: A Novel Management of Malignant Pleural Effusion

**DOI:** 10.3389/fimmu.2021.760683

**Published:** 2021-12-13

**Authors:** Xinying Li, Guannan Wu, Cen Chen, Yuan Zhao, Suhua Zhu, Xincui Song, Jie Yin, Tangfeng Lv, Yong Song

**Affiliations:** ^1^ Department of Respiratory and Critical Care Medicine, Jinling Hospital, Nanjing University School of Medicine, Nanjing, China; ^2^ Department of Respiratory and Critical Care Medicine, Nanjing Drum Tower Hospital, Nanjing University School of Medicine, Nanjing, China; ^3^ Nanjing University Institute of Respiratory Medicine, Nanjing, China; ^4^ Department of Respiratory and Critical Care Medicine, Jinling Hospital, Southern Medical University (Guangzhou), Nanjing, China

**Keywords:** malignant pleural effusion (MPE), intrapleural injection, anti-PD1, cytotoxic T lymphocyte (CTL), tumor microenvironment (TME)

## Abstract

**Background:**

Malignant tumors accompanied with malignant pleural effusion (MPE) often indicate poor prognosis. The therapeutic effect and mechanism of intrapleural injection of anti-programmed cell death protein 1 (PD1) on MPE need to be explored.

**Methods:**

A preclinical MPE mouse model and a small clinical study were used to evaluate the effect of intrapleural injection of anti-PD1 antibody. The role of immune cells was observed *via* flow cytometry, RNA-sequencing, quantitative PCR, western blot, immunohistochemistry, and other experimental methods.

**Results:**

Intrathoracic injection of anti-PD1 monoclonal antibody (mAb) has significantly prolonged the survival time of mice (P = 0.0098) and reduced the amount of effusion (P = 0.003) and the number of cancer nodules (P = 0.0043). Local CD8+ T cells participated in intrapleural administration of anti-PD1 mAb. The proportion of CD69+, IFN-γ+, and granzyme B+ CD8+ T cells in the pleural cavity was increased, and the expression of TNF-α and IL-1β in MPE also developed significantly after injection. Local injection promoted activation of the CCL20/CCR6 pathway in the tumor microenvironment and further elevated the expression of several molecules related to lymphocyte activation. Clinically, the control rate of intrathoracic injection of sintilimab (a human anti-PD1 mAb) for 10 weeks in NSCLC patients with MPE was 66.7%. Local injection improved the activity and function of patients’ local cytotoxic T cells (CTLs).

**Conclusions:**

Intrapleural injection of anti-PD1 mAb could control malignant pleural effusion and the growth of cancer, which may be achieved by enhancing local CTL activity and cytotoxicity.

**Graphical Abstract:**
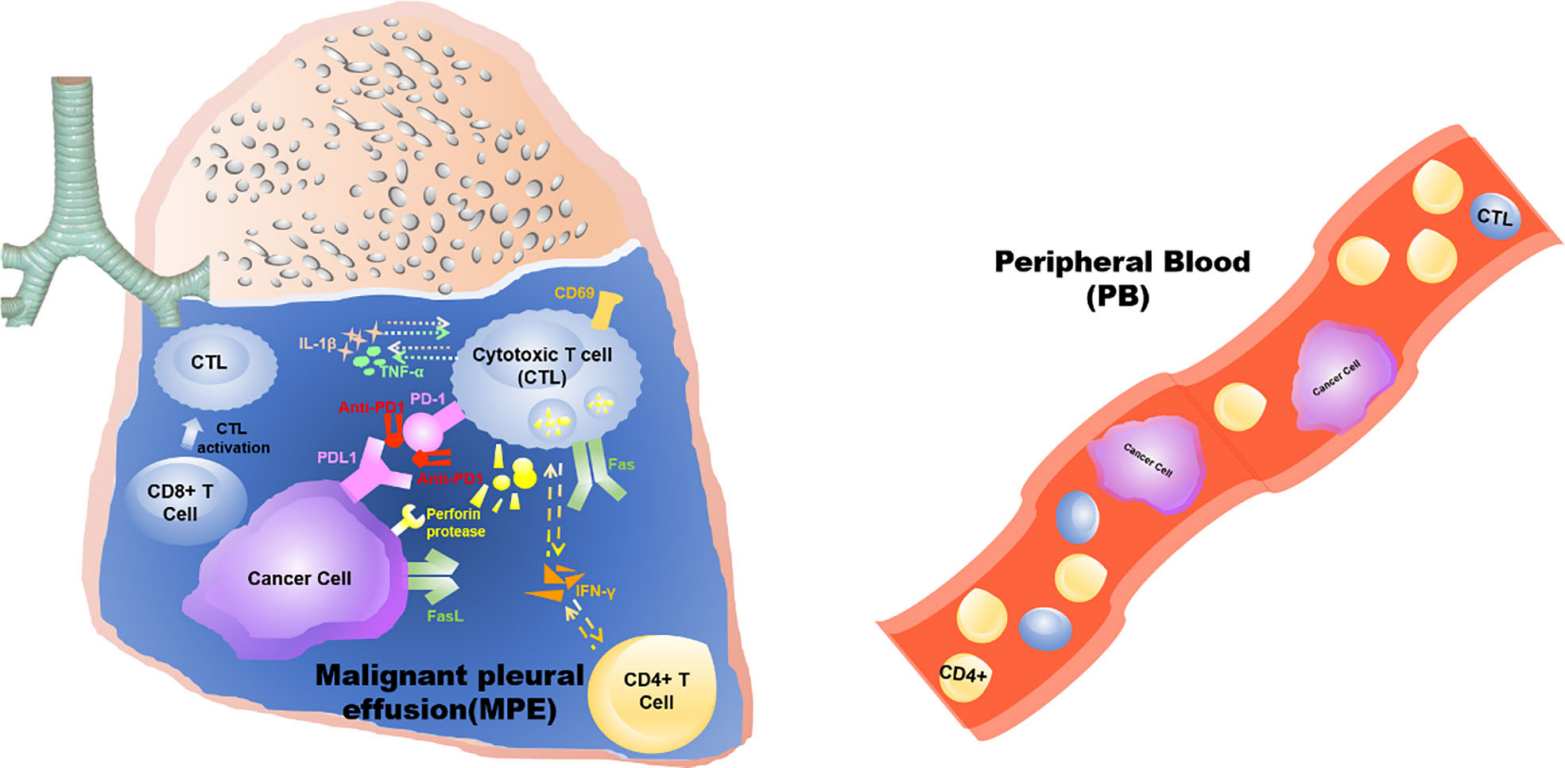
This study demonstrates that the anti-tumor effects triggered by intrathoracic injection of anti-PD1 antibody are confined to the “local” tumor microenvironment of the pleural cavity, rather than a systemic effect.

## Background

Malignant pleural effusion (MPE) refers to pleural effusion caused by the metastasis of primary pleural tumor or other malignant tumor to the pleura (1). Approximately one-third of MPEs are attributed to non-small cell lung cancer (NSCLC) (2). The median survival of MPE patients is less than 1 year (3). Combined presentation with NSCLC reduced the median survival of MPE patients to 74 days (60–92 days) (4). MPE patients often experience symptoms including shortness of breath, chest tightness, asthma, and cough due to a large amount of effusion and massive primary cancer lesions (5). The European Respiratory Society (ERS/EACT) (6) and the American Thoracic Society have both issued new MPE management guidelines (7) recommending immediate pleural puncture for patients with large effusions (7). This is the simplest and most important treatment for MPE. However, this has the disadvantage of easily causing recurrence and also leads to frequent visits to the hospital (3). To ensure long-term relief of pleural effusion symptoms (8), experts currently advocate the use of talc (9) and other local pleural intervention therapy (10) for MPE patients (11), even if patients with malignant tumors have received systemic treatment.

However, the medical talc powder that can be injected into the chest cavity has not been produced and sold in China; it is only available as a product for external use. Clinically, to reduce the recurrence of pleural effusion, the current view is that systemic treatment combined with local intervention in the pleural cavity (12) is also in line with ethical treatment. Intrapleural injection of cisplatin (13), IL-2 (14), and other treatments for MPE had cases to follow. Our team demonstrated that endostar could reduce MPE by inhibiting angiogenesis and lymphangiogenesis (15). Chloroquine has also been determined to inhibit tumor growth in animal models to lessen the production of MPE (16). Currently, there is no consensus on the local intervention scheme for MPE.

The environment of MPE is highly similar to that of the tumor microenvironment (17). The formation of effusion is related to the imbalance of a number of immune cell varities (18), and chemokine-induced inflammatory cell changes have also been determined to increase the amount of pleural effusion (19, 20). Blockage of the PD-1/programmed-death ligand 1(PD-L1) pathway has been shown to restore the interrupted antitumor immune responses (21). We have observed that expression of PD-L1 in pleural effusion cell blocks of NSCLC patients with MPE was higher than that of non-MPE patients (unpublished). A trend of longer overall survival was observed in MPE patients with PD-L1 TPS < 50% than those with TPS ≥ 50% (20.0 vs. 13.8 months) (22). At present, there are no reports describing the use of immune checkpoint inhibitors for local treatment of MPE. Therefore, there is an urgent requirement to explore whether intrapleural injection of anti-PD1 monoclonal antibody (mAb) can treat MPE.

## Materials And Methods

### MPE Animal Model Establishment and Treatment Schedule

Mouse Lewis lung carcinoma cell line (LLC) was purchased from Shanghai Cell Bank of Chinese Academy of Sciences. The MPE mouse model (C57BL/6J, six-week-old, male; Model Animal Research Center of Nanjing University) has been previously described (18, 20) **(** **Figure S1** **)**. Mice were reportedly administered with an intrathoracic injection of 200μg anti-PD1 mAb (InVivoMab anti-mouse PD-1 (CD279), Bioxcell)/0.9% normal saline on the 7th and 14th day of the model. We then injected 50 μL anti-CD8 mAb (4 mg/kg, InVivoPlus anti-mouse CD8A, Bioxcell) intraperitoneally multiple times to achieve CD8 depletion. The fixed mouse tissues were successively scanned using a CT machine (Siemens Somatom Sensation 16).

### Patients and Assessments

From September 2019 to March 2020, nine advanced NSCLC patients with MPE who were hospitalized in the Department of Respiratory and Critical Care, Jinling Hospital, Nanjing University School of Medicine, were recruited for this clinical study. The research was approved by the Ethical Committee and Institutional Review Board of the Jinling Hospital, which is affiliated to Nanjing University School of Medicine (Ethical code:2018NZKY-031-03). All patients were assessed by CT and then underwent thoracic puncture drainage (less than 100 ml/d, for 2 consecutive days). IBI308 (sintilimab,100 mg) was then diluted in normal saline and injected after adequate drainage. Patients were required to return to the hospital for chest CT review at 5 and 10 weeks after discharge for assessment. NCI-CTC AE version 4.03 standard was used to evaluate the safety of drugs. Pleural fluid was collected from all patients before and after treatment.

### Flow Cytometry

Cancerous nodules in the thoracic cavity of mice were collected after dissection and digested with the addition of Ca^2+^ collagenase (Sigma–Aldrich). Lymphocytes were isolated from the patient’s pleural effusion using blood density gradient centrifugation. The following antibodies were used: fixable viability dye eFluor 780, Alexa Fluor^®^488 anti-mouse CD3 antibody, mouse CD3E PERCP mAb 0.025 mg 145-2C11, PE/Cy7 anti-mouse CD45 antibody, FITC anti-mouse CD8b (Ly-3) antibody, APC anti-mouse CD69 antibody, Alexa Fluor^®^647 anti-mouse CD4, PE anti-mouse IFN-γ antibody, APC anti-human/mouse granzyme B recombinant antibody, APC-Cy™7 mouse anti-human CD3, FITC mouse anti-human CD8, FITC mouse anti-human CD4, PerCP mouse anti-human CD69, and PE mouse anti-human CD279.

### RNA-Sequencing (RNA-Seq) and Quantitative PCR (qPCR)

RNA extraction from tumor tissues in the mouse pleural cavity was performed using TRIzol™ Reagent (Invitrogen). RNA purity (NanoPhotometer) and concentration (Qubit 2.0 Fluorometer) were determined, and RNA integrity was then assessed (Agilent 2100 Bioanalyzer). The Illumina ^®^ UltraTM RNA Library Prep Kit was used to construct the library. Gene Ontology (GO) enrichment analysis and Reactome database analysis were performed. qPCR (Takara SYBR Premix Ex TaqTM) experiment was performed on a qPCR instrument (Quantagene q225). Primer sequences are shown in **Supplementary Table 1**.

### ELISA, Western Blot, Immunohistochemistry (IHC), and LDH Release Experiment

The levels of TNF-α and IL-1β (Novus Biologicals, Inc.) in pleural effusions and the blood of mice were determined. Anti-CD8 antibody (Abcam), anti-PD1 antibody (EPR20665, Abcam), anti-PDL1 antibody (EPR20529, Abcam), anti-GAPDH antibody (ab8245, Abcam), and anti-β-actin antibody (13E5, CST) were used for antigen detection. Lymphocyte and tumor cell lines were co-cultured in 96-well plates at a ratio of 20:1. The LDH release experiment was then performed using the lactate dehydrogenase cytotoxicity test kit (Beyotime Biotechnology).

### Statistical Analysis

After collecting the clinicopathological data of the patients, statistical classification was summarized by using Excel. Flow cytometry results were re-aggregated and analyzed using FlowJo. SPSS and GraphPad prism 5.0 were used for statistical plotting. All experiments were performed with ≥ 3 biological replicates. The independent sample t-test was used to compare data between two groups. *P < 0.05, **P < 0.01, ***P < 0.001 were considered to be of significant difference, whereas ns showed no significant difference.

## Results

### Decreased MPE in Mice After Intrapleural Injection of Anti-PD1 mAb

**Figure 1:**
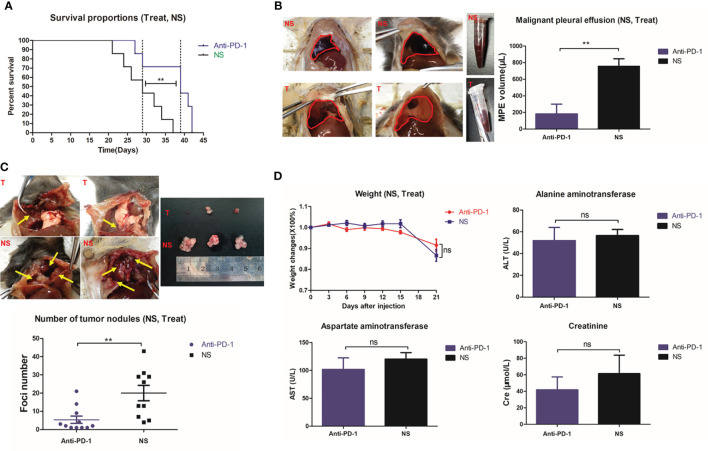
Intrathoracic injection of Anti-PD1 monoclonal antibody could effectively control MPE in mice. **(A)** Kaplan-Meier survival curve showed that the overall survival of mice receiving Anti-PD1 treatment (blue) was significantly prolonged. **(B)** Two groups of mice with MPE were photographed; Histogram of the amount of MPE in the treatment group (blue) and the control group (black). **(C)** Cancerous nodules were seen in the pleural cavity of two groups of mice; Comparison of scatter plots of cancer nodules in the treatment group (blue) and the control group (black). **(D)** The fold chart of weight change at day 3, 6, 9, 12, 15 and 21 after modeling; Levels of ALT (U/L), AST (U/L) and Cre (μmol/L) in the blood were compared after treatment in the two groups. *P < 0.05; **P < 0.01, ***P < 0.005; ns, P > 0.05.

The median survival of MPE model mice treated with intrapleural injection of anti-PD1 mAb was approximately 1.345-fold compared to that of the control mice treated with 0.9% saline (39 days vs. 29 days, log-rank p = 0.0098) **(** **Figure 1A** **)**. In addition, the average volume of pleural effusion in the anti-PD1 mAb group was less than 300 μL and that in the NS group was more than 500μL (P=0.003) **(** **Figure 1B** **)**. The size and number of cancerous nodules in the treatment group were determined to be lower than those in the NS group (P = 0.0043) **(** **Figure 1C** **)**. Although both groups experienced dramatic weight loss at the later stage of the model, no significant difference was detected **(** **Figure 1D** **)**. The levels of alanine aminotransferase (ALT), aspartate aminotransferase (AST), and creatinine (Cre) in the blood of mice did not vary significantly **(** **Figure 1D** **)**, suggesting that no obvious toxic or side effects were observed when intrapleural injection of anti-PD1 mAb was administered.

### Intrapleural Injection of 200μg Anti-PD1 Mice Produced the Longest Period of Survival

**Figure 2:**
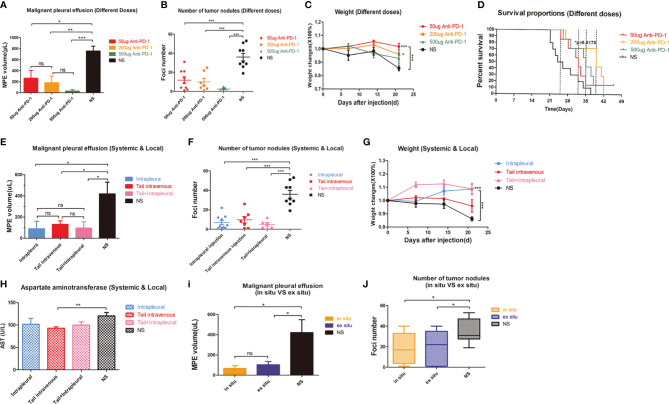
Intrapleural administration had little effect on liver enzymes. **(A)** The average MPE amount of mice in different dose groups. **(B)** The number of tumor nodules in the thoracic cavity of mice in different dose groups. **(C)** Weight change of mice in different dose groups (T test was used for comparison between two groups, and two-factor analysis in multiple groups was used for 2way ANOVA analysis of variance). **(D)** Kaplan-Meier survival curves of mice in different dose groups. **(E–G)** Comparison of MPE amount, number of cancerous nodules and weight change in each group of mice with different drug administration methods. **(H)** AST (U/L) level in mice after tail vein injection administration. **(I)** MPE amount and **(J)** number of tumor nodules in the contralateral and weight changes *in-situ* and NS groups were compared. *P < 0.05; **P < 0.01, ***P < 0.005; ns, P > 0.05.

Intrapleural injection of 500μg high-dose anti-PD1 mAb was the most effective dose for MPE (P = 0.0008) **(** **Figure 2A** **)**; it also limited the proliferation of cancer nodules to the greatest extent (P = 0.0003). However, even one-tenth (50μg) of the administered dose could also limit the amount of MPE and the number of cancerous nodules **(** **Figure 2B** **)**. The weight of mice in the high-dose group (500μg) was observed to decrease gradually from the second week **(** **Figure 2C** **)**, considering the obvious side effects. Log-rank analysis showed that each dose group had an extended survival period (P = 0.0178) **(** **Figure 2D** **)**. Mice treated with 200μg anti-PD1 mAb achieved the longest median survival period.

Interestingly, intrathoracic and caudal intravenous injections yielded similar results (**Figures 2E, F**). Local administration (**Figures S2A, B**) compensated for the issues with poor safety with systemic administration **(** **Figure 2G** **)**. Systemic administration was determined to have a greater impact on AST than intracavitary administration (P=0.0069) **(** **Figure 2H** **)**. Surprisingly, contralateral intrapleural administration **(** **Figure S2C–F** **)** could also reduce the amount of MPE produced in mice **(** **Figure 2I** **)** and the number of cancerous nodules **(** **Figure 2J** **)**.

### Local CD8+ T Cells Participated in Intrapleural Administration of Anti-PD1 mAb

**Figure 3:**
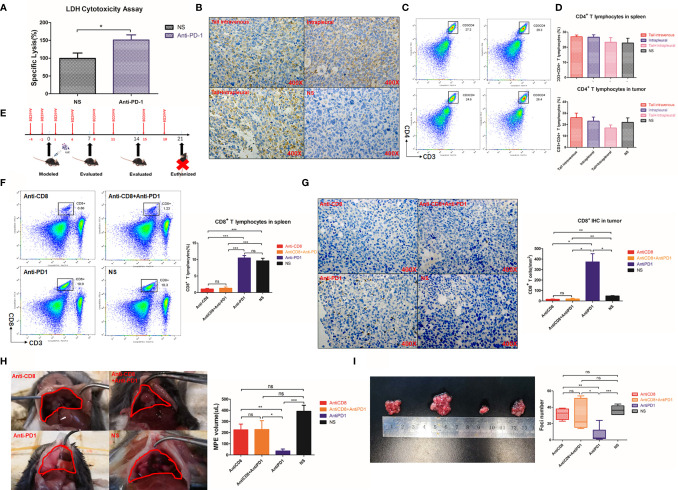
The therapeutic effect of Anti-PD1 mAb in MPE is mainly related to local CD8 + T cells. **(A)** LDH release experiment was used to detect the mortality of LLC after co-culture of lymphocyte-LLC in Anti-PD1 treatment group and NS group. **(B)** Immuno-histochemical staining (400X) of CD8 in tumors of mice through tail vein injection, intrapleural injection, tail + intrapleural injection of Anti-PD1 mAb and NS group. **(C)** Flow cytometry was used to observe CD3+CD4 + T cell population in the spleen of systemic therapy, local therapy, combined therapy, and NS group. **(D)** The histogram showed the proportion of CD3 + CD4 + T cells in all lymphocytes in the spleen and tumors of each group. **(E)** According to the body weight (g) of mice, CD8 monoclonal antibody (4mg/kg) was continuously given to mice for “CD8 depletion” modeling. Mice were injected intraperitoneally with CD8 mAb on days -4, -1, 1, 4, 8, 11, 15, and 18 of MPE modeling. The mice were evaluated every week and were sacrificed on the 21st day. **(F)** Flow cytometry was used to observe the proportion of CD8 + T cells in the spleens of Anti-CD8 group, Anti-CD8 + Anti-PD1 group, Anti-PD1 group and NS group. **(G)** IHC results (400X) of CD8 in mice tumors of Anti-CD8 group, Anti-CD8 + Anti-PD1 group, Anti-PD1 group and NS group. Randomly count the number of ≥3 positive cells in 200X field of view. **(H)** Photographs of the diaphragm of the above four groups of mice (circled in red) and histogram of MPE amount. **(I)** Photographs of cancer nodules and counts of cancer nodules in the chest of the above four groups. *P < 0.05; **P < 0.01, ***P < 0.005; ns, P > 0.05.

Lymphocytes were involved in treating MPE with anti-PD1 mAb **(** **Figure 3A** **)** and accounted for higher local CD8+ T cell infiltration **(** **Figure 3B** **)**. Flow cytometry showed that the proportion of CD3+ CD4+ T cells in the spleen of mice treated with the different methods was maintained at 20%–30% **(** **Figure 3C** **)**. There was also no difference in the proportion of CD3+ CD4+ T cells in tumors of these mice **(** **Figure 3D** **)**.

CD8 depletion in mice was achieved by continuous intraperitoneal injection of anti-CD8 antibody **(** **Figure 3E** **)**, resulting in a sharp drop of CD8+ T cells in the spleen to less than 10% of the original level (P < 0.001) **(** **Figure 3F** **)**. The CD8 depletion group obtained the lowest number of CD8+cells. There was no fluctuation of local CD8 infiltration even after anti-PD1 treatment **(** **Figure 3G** **)**. The mice in the anti-PD1 group had the least amount of MPE. However, despite simultaneous clearance of CD8 and immunotherapy, there was no improvement in controlling excessive MPE **(** **Figure 3H** **)**. The number of cancer nodules remained significant (P = 0.0013) after CD8 depletion, and subsequent anti-PD1 treatment did not resolve the substantial number of cancerous nodules in the chest (P = 0.0187) **(** **Figure 3I** **)**.

### Local Cytotoxic T Lymphocyte (CTL) Antitumor Effect Was Enhanced After Intrapleural Injection of Anti-PD1 mAb

**Figure 4:**
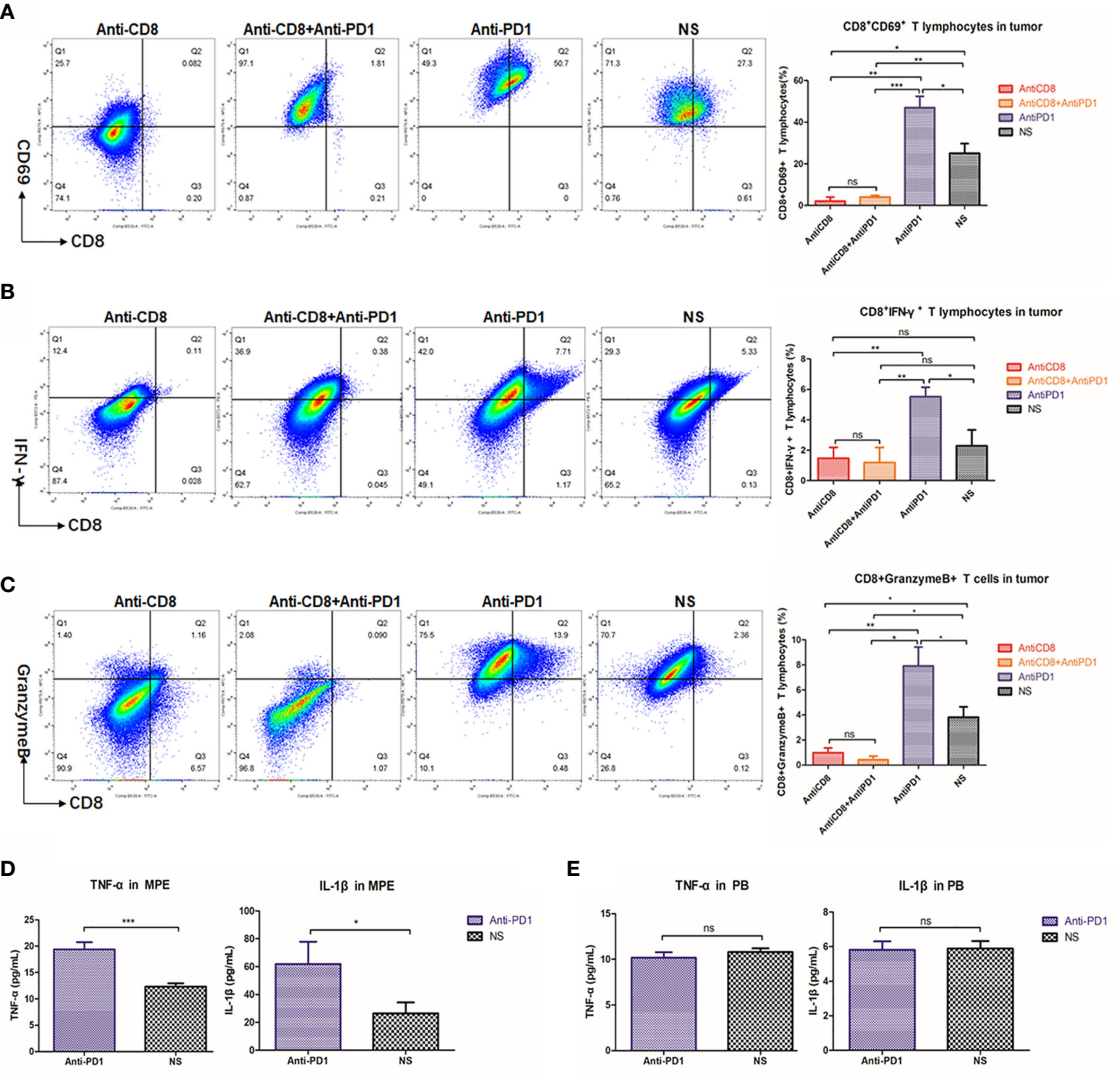
Anti-PD1 treatment of MPE could increase the local anti-tumor response of CTL. **(A)** Density map showed the CD8 + CD69 + cell population in tumors of Anti-CD8 group, Anti-CD8 + Anti-PD1 group, Anti-PD1 group and NS group. The histogram showed the proportion of CD8+ CD69+ T cells in these four groups of local cancers in all lymphocytes. **(B)** The percentage of CD8+IFN-γ+ cells in all lymphocytes was compared among the four groups of local cancers. **(C)** The ratio of Granzyme B-releasing CD8 + T cells in all the lymphocytes among the four groups of local cancers. **(D)** ELISA was used to determine the release levels of TNF-α and IL-1β from the supernatant of mouse pleural effusion. **(E)** The levels of TNF-α and IL-1β released from the peripheral blood supernatant (plasma) of mice. *P < 0.05; **P < 0.01, ***P < 0.005; ns, P > 0.05.

CD69 (23) and intracellular factor IFN-γ are both important markers reflecting the activation of CD8+ T cells (24). Due to the absence of CD8, the number of CD8+ T cells expressing CD69 in pleural cavity was also extremely low **(** **Figure 4A** **)**. Intrapleural injection with anti-PD1 after CD8 depletion did not prevent the weakened IFN-γ secretion by CTLs (P = 0.0064) **(** **Figure 4B** **)**. The activity of CD8+T cells at the tumor site was most enhanced in the anti-PD1 mAb treatment group, *via* intrapleural injection, compared with those of other groups (P = 0.0337). Using flow cytometry, we observed that the proportion of CD8+ T cells releasing granzyme B peaked in the tumors of mice receiving anti-PD1 treatment **(** **Figure 4C** **)**. In the spleen, after CD8 depletion, IFN-γ and granzyme B secreted by CD8+ T cells were not much **(** **Figure S3** **)**. In the pleural effusion of mice receiving intrapleural injection, the levels of TNF-α (P = 0.0004) and IL-1β (P = 0.0422) were significantly higher than those of the NS group **(** **Figure 4D** **)**. However, the concentration of these factors in the peripheral blood of mice did not differ before or after treatment **(** **Figure 4E** **)**. Local anti-PD1 treatment failed to change the levels of systemic inflammatory factors in mice.

### Local Tumor Microenvironment (TME) Was Affected by Intrapleural Injection of Anti-PD1 mAb

**Figure 5:**
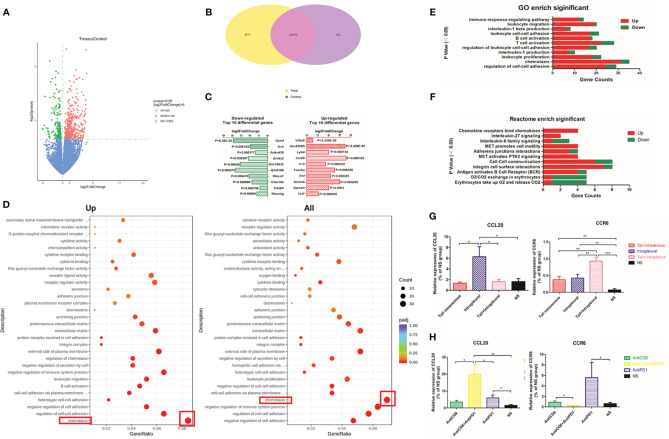
Differentially expressed genes enriched in the chemokine family after local Anti-PD1 treatment. **(A)** The volcano map compared the differential gene distribution of the two groups with or without Anti-PD1 treatment. The abscissa indicates the fold change of gene expression in the treatment group and the control group (log2FoldChange), and the ordinate indicates the significance level of the gene expression difference between the two groups (-log10padj or -log10pvalue). Up-regulated genes are indicated by red dots and down-regulated green. **(B)** Wayne graph shows the number of differential genes shared between the treatment group and the NS group. **(C)** The bar chart lists the top 10 genes that were most significantly up-regulated (red on the right) and down-regulated (green on the left) in the treatment group than in the NS group. **(D)** Scatter plots were used to map the 30 most significant pathways in GO analysis of up-regulated (left) and all (right) differential gene enrichment. GeneRatio is the ratio of the number of different genes to the total number of different genes. The ordinate lists the name of the GO pathway, and the size of the dots represents the number of genes in each pathway. The shade of color represents saliency. **(E)** Immune-related pathways significantly enriched in GO analysis. The P values are arranged from small to large from top to bottom. The abscissa is the number of annotated genes (red up, green down), and the ordinate is the GO Term name. **(F)** Some Reactome pathways related to immunity. **(G)** The expression level of CCL20 and CCR6 mRNA in tumor tissue of systemic administration, local administration, combined administration and NS group. **(H)** CCL20 and CCR6 mRNA expression levels in AntiCD8, AntiCD8 + AntiPD1, AntiPD1, NS group. *P < 0.05; **P < 0.01, ***P < 0.005; ns, P > 0.05.

In total, 420 genes were upregulated and 189 downregulated (red and green, respectively, in volcano plot, **Figure 5A**) in the treatment group compared with those in the NS group. The Venn diagram showed that these two groups co-expressed more than 120,000 genes, of which 970 were unique in the treatment group and 442 were unique in the control group **(** **Figure 5B** **)**. After local anti-PD1 treatment, a large number of genes including human latent-transforming growth factor beta-binding protein 2 (*Ltbp2*) and lymphocyte antigen 6 complex (*Ly6D*), and chemokine ligand 20 (*Ccl20*) were rapidly upregulated; while the expression of the programmed-death receptor gene Pdcd1(P = 0.031), granzyme F (*Gzmf*), *Hba-a1*, and *Clec10a*, etc. were all significantly down-regulated **(** **Figure 5C** **)**.

In the GO and Reactome enrichment analysis of upregulated differential genes, the genes annotated to the chemotaxis pathway were the most numerous with the greatest significant difference in expression levels **(** **Figures 5D–F** **)**. CCL20 is the ligand of CCR6 on the surface of immune cells (B cells, T cells, immature DC) (25). The mRNA level of *Ccl20* with intrapleural injection of anti-PD1 mAb was 3.84-fold higher than that of the control group (P = 0.0303); additionally, the level of *Ccr6* mRNA was also significantly increased (P = 0.0074) **(** **Figure 5G** **)**. Following CD8 clearance, expression of *Ccl20* mRNA was slightly lower than that of the treatment group; however, expression of *Ccl20* increased with both CD8 clearance and local anti-PD1 treatment. The expression of *Ccr6* in the treatment group was highest (P = 0.0373), whereas this did not increase in the CD8-cleared group **(** **Figure 5H** **)**.

**Figure 6:**
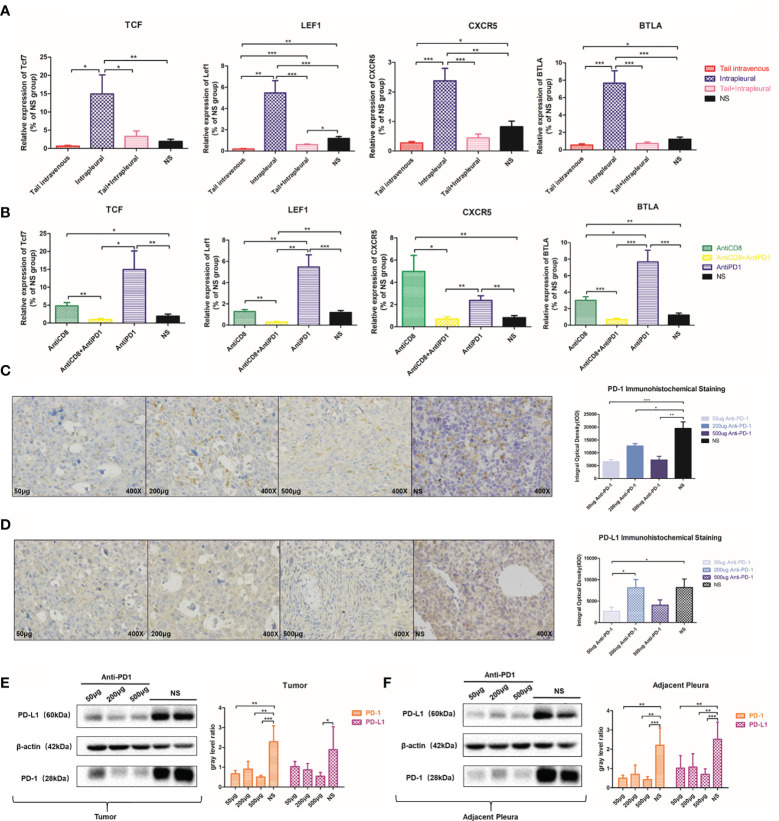
Local anti-PD1 treatment affected the expression of immune-related molecules. **(A)** Tcf7, LEF1, CXCR5, and BTLA mRNA expression levels of tumors in the thoracic cavity of the systemic, local, co-administered, and NS groups. **(B)** The expression levels of Tcf7, LEF1, CXCR5, and BTLA genes in AntiCD8, AntiCD8 + AntiPD1, AntiPD1, and NS group. **(C)** 50μg, 200μg, 500μg Anti-PD1 treatment group and NS group MPE model mice PD-1 immunohistochemistry in the chest cavity. The histogram compared the PD-1 integral optical density of tumor nodules in the chest cavity of mice in different doses group. **(D)** Comparison of PD-L1 IHC results and optical density values in the above four groups. **(E)** Western Bolt was used to detect the PD-1 protein level in 50μg, 200μg, 500μg Anti-PD1 treatment group and NS group tumor in the thoracic cavity of mice, and the gray value was compared. **(F)** PD-L1 protein expression in the thoracic cavity of the above four groups of mice. All experiments were performed with ≥3 biological replicates. The independent sample T test was used to compare data between two groups. *P < 0.05, **P < 0.01, ***P < 0.001 were considered statistically significant.

CXCR5 (26), TCF7, LEF1, and BTLA are all involved in T lymphocyte activation pathway (27). After anti-PD 1 treatment, *Tcf7* (P = 0.0092), *Lef1* (P = 0.0007), and *Btla* (P = 0.0002) mRNA expression increased more than 4 times. Expression of *Cxcr5* in the control group was only 34.4% compared to that of the treatment group (P = 0.028) **(** **Figure 6A** **)**. There was significant inhibition of expression of *Tcf7*, *Lef1*, and *Btla* mRNA following CD8 depletion, and anti-PD1 therapy has increased this effect **(** **Figure 6B** **)**.

Interestingly, with IHC staining of cancerous nodules on the pleura of different groups of mice, PD-1 staining in the low-dose group (P < 0.0001), the medium-dose group (P = 0.0419), and the high-dose group (P = 0.0021) was not as clear as that in the NS group **(** **Figure 6C** **)**. PD-L1 expression was also suppressed to a certain extent after receiving anti-PD1 treatment **(** **Figure 6D** **)**. We verified *via* western blots that PD-1 and PD-L1 protein expressions in the treatment group were significantly inhibited in adjacent tissues **(** **Figure 6E** **)** and tumor tissues **(** **Figure 6F** **)** in comparison with those in the control.

### Intrapleural Injection of Anti-PD1 mAb Had a Favorable Effect on NSCLC Patients With MPE

**Figure 7:**
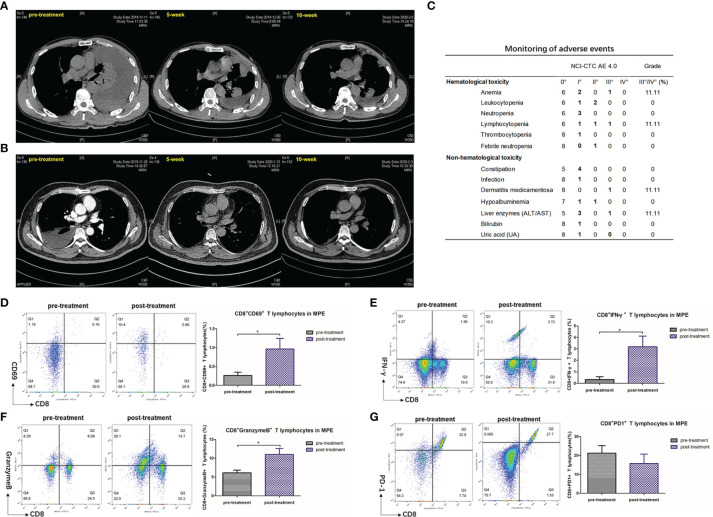
Pleural effusion in lung cancer patients with MPE decreased after intrapleural injection of Anti-PD1 mAb. **(A)** The patient is a 51-year-old male who has been receiving a systemic treatment regimen of Pemetrex + Nedaplatin since his diagnosis of adenocarcinoma (T4N0M1c). The baseline length of the lesion was 47mm. Chest CT recorded about 4000 ml of fluid in the left chest before receiving local Anti-PD1 treatment. On the fifth week after administration, he was returned to the hospital for evaluation of the lesion with a diameter of 44 mm, and no pleural effusion was drained. The effusion did not drain out at 10 weeks after treatment, and the length of the lesion was 49 mm. The condition of the two assessments was SD, maintaining the original systemic treatment plan. During treatment, there was a decrease in lymphocyte count (CTCAE II °) and recovery after receiving granulocyte stimulating factor. **(B)** A 55-year-old male adenocarcinoma patient with EGFR 19 exon deletion mutation (T2bN1M1c). After the diagnosis, the patient received 1 chemotherapy (Pemetrex+ Carboplatin), and then began oral administration of Osimertinib for systemic treatment. Five months after the diagnosis, the patient first found that the right pleural effusion was about 2500m. The pleural effusion almost disappeared after 5 weeks of pleural administration, and the effusion could not be drained after 10 weeks. The baseline of the focal length of the lesion was 34 mm, and the condition was stable in the subsequent two evaluations, with no change in the focal length. During the treatment, the patient had experienced drug-induced dermatitis (CTCAE III), which was relieved after receiving symptomatic hormone therapy. **(C)** Monitoring of adverse reactions in 9 lung cancer patients with MPE. **(D–G)** Flow analysis of CD8+ CD69+ cell population, CD8+ IFN-γ+ cell population, CD8+ Granzyme B+ cell population, CD8+ PD-1+ in MPE before and after intrapleural injection of Anti-PD1. Independent sample T test. *P < 0.05; **P < 0.01, ***P < 0.005; ns, P > 0.05.

The clinicopathological characteristics of the nine advanced NSCLC patients with MPE included in this study are summarized in the table below **(** **Table 1** **)**. After adequate drainage, we injected human anti-PD1 (sintilimab) into the pleural cavity, and its effectiveness and safety in treating MPE were then evaluated **(** **Figures 7A, B** **)**. After 5 weeks of intrapleural administration, pleural effusion volume decreased substantially in seven out of nine patients. Following a further 10 weeks of intrapleural administration, the control rate of pleural effusion was still 66.7% **(** **Table 1** **)**. Adverse reactions were rare in these patients, with grade III adverse reactions occurring in only a few cases **(** **Figure 7C** **)**. After treatment, consistent with the results of animal experiments, there was a significant increase in the levels of CD69 (P = 0.0391), a molecule closely related to CD8+ T lymphocyte activation **(** **Figure 7D** **)**, and IFN-γ, which is secreted by CTLs, (P = 0.0252) **(** **Figure 7E** **)**. The proportion of CD8+ T cells positive for molecular granzyme B also has significantly increased following intrapleural injection of anti-PD1 mAb (P = 0.0187) **(** **Figure 7F** **)**. The expression of PD-1 on the surface of CTL in MPE showed a decreasing trend **(** **Figure 7G** **)**. The activity and killing capacity of CD8+ T cells in peripheral blood were noted to increase, but lacked significant difference **(** **Figure S4** **)**.

**Table 1:** Clinical characteristics of MPE patients.

Characteristics	No. of patients	(%)
Age (Years)		
Median	62	
Range	51-75	
Gender		
Male	7	77.8
Female	2	22.2
ECOG Score		
1	9	100
Smoking history		
Yes	2	22.2
No	7	77.8
Targeted mutation		
Positive	5	55.6
Negative	4	44.4
Extra-pleural metastasis		
Yes	4	44.4
No	5	55.6
Systemic treatment		
Pemetrexed+Platinum	4	44.4
Targeted drugs	2	22.2
No	3	33.3
5-week pleural fluid control rate		
Yes	7	77.8
No	2	22.2
10-week pleural fluid control rate		
Yes	6	66.7
No	3	33.3

## Discussion

Delaying the recurrence of malignant pleural effusion and improving survival have always been problems that need to be overcome, and there is no uniform standard in the industry. For the first time, our team attempted to improve MPE by intrathoracic injection of immune checkpoint inhibitors. Here, the median survival time of the mice in the treatment group was 10 days longer than that in the control group. Intrapleural injection of anti-PD1 mAb was demonstrated to have a therapeutic effect on MPE in mice.

Intraperitoneal injection of a localized low-dose anti-CTLA-4 mAb has controlled the growth of colorectal cancer in mice and was not inferior to the administration of a systemic high-dose treatment (28). Our study reached a similar conclusion regarding the management of MPE. Intrapleural injection of anti-PD1 therapy is a favorable alternative to systemic administration and has minimal systemic effect. The control of MPE and nodules in the contralateral pleural cavity was a surprising result. However, we considered that it was related to bilateral pleural effusion caused by extensive metastasis of primary carcinoma.

We first observed extensive infiltration of CD8+ T cells in the tumor area after intrapleural injection. After CD8 removal, the effect of anti-PD1 treatment group was decreased, which further explained the critical role of CD8+ T cells in treating MPE with anti-PD1 mAb. Due to the activation effect of CD8+ T cells, CTLs are required to produce a lasting and effective antitumor immune response in MPE (29). We found that the proportion of CD8+ CD69+ and CD8+ IFN-γ+ T cells in the treatment group was significantly higher than that in the NS group. After CD8 clearance, low localized CTL activation could not be corrected, even if anti-PD1 treatment was received afterward. These results show that CTLs that infiltrated into the local (cancer area) of mice were activated. The perforin/granzyme pathway is one of the systems used by CTLs to kill tumor cells (29). The increase in the number of CD8+ T cells releasing granzyme B is an evidence of improved killing function of CTLs in the local pleural cavity. There was no significant difference in the ratio of CTL expressing CD69 and IFN-γ in the spleen of mice, and the ratio of CD8+ granzyme B + cell population was relatively stable. Therefore, we believe that local immunotherapy has little effect on the activity and function of systemic CTLs.

We have also examined the effect of treatment on two pro-inflammatory factors, TNF-α and IL-1β. The use of combined immunotherapy, including anti-PD1 therapy, can enhance the release of TNF-α and other factors from T cells to induce stronger and more effective antitumor effects (30). In our experiments, the large increase in TNF-α and IL-1β levels in the pleural fluid after intrapleural injection can be attributed to the bystander effect (30). However, there was no increase in the levels of pro-inflammatory cytokines in the peripheral blood of mice before and after treatment, which we believe is consistent with the results of flow cytometry analysis of mouse spleen cells. We speculate that the antitumor effect of intrapleural injection is limited to the local environment of the pleural cavity, rather than being systemic.

MPE, like the immune microenvironment of other malignancies, has a complex interplay of cells and molecules (17). The role of the CCL20/CCR6 pathway in tumor immunity is a controversial topic in light of current known studies. For instance, CCR6/CCL20 is involved in the occurrence and progression of tumors (31). However, some researchers believe that this pathway is also involved in the restoration of antitumor immune effects (32). Here, the CCL20/CCR6 pathway was clearly activated after treatment with anti-PD1 mAb in the local pleural cavity but remained at a low level in the CD8 clearance group. Thus, intrapleural injection may promote local infiltration of CTLs through the activation of CCL20/CCR6 pathway in the local microenvironment, but the role of immune cell recruitment, such as DCs, remains to be explored.

CXCR5 has been identified as the receptor for CXCL13, which is expressed in T- follicular helper cells (Tfh) (33) and follicular cytotoxic T cells (Tfc) (27). The deletion of the genes *Lef1* and *Tcf7*, which encode the transcription factors LEF-1 (34) and TCF-1 (35), would then cause defects in T cell development, including that of CD8+ T cells (36). The levels of *Cxcr5*, *Tcf7*, and *Lef1* mRNA were all significantly increased after intrapleural injection, indicating that the local treatment with anti-PD1 mAb may affect the activation of Tfh and Tfc cells in the local microenvironment. PD-1 and BTLA are evolutionarily related and are co-expressed on human and mouse tumor antigen-specific CD8+ T cells (37). The explanation for the high expression of *Btla* gene is that the function BTLA can substitute for that of PD-1.

Both IHC and western blot data show that the expression of PD-1 in intrapleural cancer and adjacent cancer areas was significantly reduced, which may be due to the activation of PD-1 insufficient expression induction (38). A significant decrease in the expression of PD-L1 in local and adjacent cancers was also observed. We hypothesized that the expression of PD-L1 might be closely related to the response (39) after immune checkpoint inhibitor treatment. The resistance of some patients to immunotherapy may be related to the decreased expression of PD-L1.

Sintilimab (a human anti-PD1 mAb) has been approved for second-line systemic treatment in lymphoma by the National Medical Products Administration of China, and it has demonstrated advantages in treating NSCLC [ORIENT-11 (40)]. The control rate of pleural effusion in NSCLC patients with MPE after 5 weeks of intrapleural injection of sintilimab was 77.8%. The results of low cytometry analysis of samples from pleural effusion in patients were consistent with the results of animal experiments, which verified the improved activity and function of CTLs in the local pleural cavity.

At present, our hypothesis of local administration and experimental design are still relatively new attempts in the clinical tumor field of immunotherapy for MPE. We comprehensively considered this hypothesis first in a single-arm exploratory study, and the efficacy of the 9 patients included in the study was acceptable. Of course, we will also try to carry out a larger sample size multi-center study in this direction under the condition of ethical approval, and design a randomized controlled test to verify this hypothesis.

From the point of view of economic cost, it is well known that the most commonly used pleurodesis is talc, the price is very cheap. However, medical talc, which can be injected into the chest, is not yet produced and sold in China. Immunotherapy is indeed more expensive than many drugs used for intraperural injection, such as cisplatin, IL-2 and endostatin. However, based on the gradual promotion and application of anti-PD1 therapy in various tumor treatments, with the gradual optimization of relevant drug production technology and the support of the government, we believe that it will become an affordable and cost-effective drug for more people in the near future.

Our study is the first to propose intrapleural injection of immune checkpoint inhibitors for treating MPE, and it was experimentally validated in animal models and clinical patients. Moreover, we preliminarily revealed the complex local tumor microenvironment of MPE. To expand on these results, the application of other immune checkpoint inhibitors in pleural diseases will be explored in future work.

## Data Availability Statement

The original contributions presented in the study are publicly available. This data can be found here: https://www.ncbi.nlm.nih.gov/bioproject/PRJNA761009.

## Ethics Statement

The studies involving human participants were reviewed and approved by The Ethical Committee and Institutional Review Board of the Jinling Hospital, affiliated to Nanjing University School of Medicine (Ethical code:2018NZKY-031-03). The patients/participants provided their written informed consent to participate in this study. The animal study was reviewed and approved by The Ethical Committee and Institutional Review Board of the Jinling Hospital, affiliated to Nanjing University School of Medicine (Ethical code:2018NZKY-031-03). Written informed consent was obtained from the owners for the participation of their animals in this study. Written informed consent was obtained from the individual(s) for the publication of any potentially identifiable images or data included in this article.

## Author Contributions

XL and YS conceived and designed the study. XL conducted most of the experiments. GW, CC, YZ, and SZ performed parts of the experiments. SZ, XS, and JY provided reagents and acquired data. XL, CC, and YZ analyzed data. XL and GW wrote the manuscript. TL and YS supervised the project and contributed to writing the manuscript. All authors contributed to the article and approved the submitted version.

## Funding

This work was supported by grants from the National Natural Science Foundation of China (grant number 81772500) and the Key Research and Development Program of Jiangsu Province (grant number BE2019719).

## Conflict of Interest

The authors declare that the research was conducted in the absence of any commercial or financial relationships that could be construed as a potential conflict of interest.

## Publisher’s Note

All claims expressed in this article are solely those of the authors and do not necessarily represent those of their affiliated organizations, or those of the publisher, the editors and the reviewers. Any product that may be evaluated in this article, or claim that may be made by its manufacturer, is not guaranteed or endorsed by the publisher.

## Abbreviations

MPE, malignant pleural effusion; NSCLC, non-small cell lung cancer; mAb, monoclonal antibody; LLC, Lewis lung carcinoma cell line; TME, tumor microenvironment; ALT, alanine aminotransferase; AST, aspartate aminotransferase; Cre, creatinine; CTL, cytotoxic T lymphocytes; Tfh, T-follicular helper cells; Tfc, follicular cytotoxic T cells.
