# Supplementary material for: Intrapleural Injection of Anti-PD1 Antibody: A Novel Management of Malignant Pleural Effusion
Source: Front Immunol. 2021 Dec 13;12:760683. doi: 10.3389/fimmu.2021.760683 (PMC8711587; doi:10.3389/fimmu.2021.760683)
Supplement: Supplementary file 1 [file DataSheet_1.pdf]

Supplementary Figures

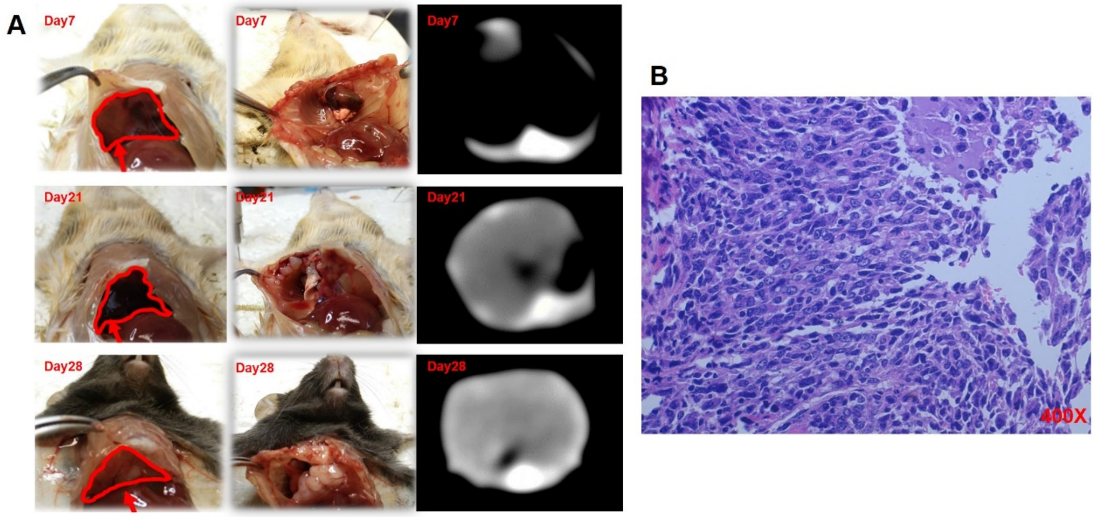

Figure S1. Establishment of a mouse model of malignant pleural effusion. (A) Mice were sacrificed on day 7, day 21, and day 28 (4 weeks) after modeling. The area marked by the red arrow is the pleural septum. The darker the color, the more bloody effusion. The distribution of cancer nodules in the pleural cavity gradually increased with the prolonged modeling time. The effusion volume is scanned sequentially by a CT machine (Siemens Somatom Sensation16). (B) Pathological sections of cancerous nodules in the pleural cavity of mice (HE staining, 400X).

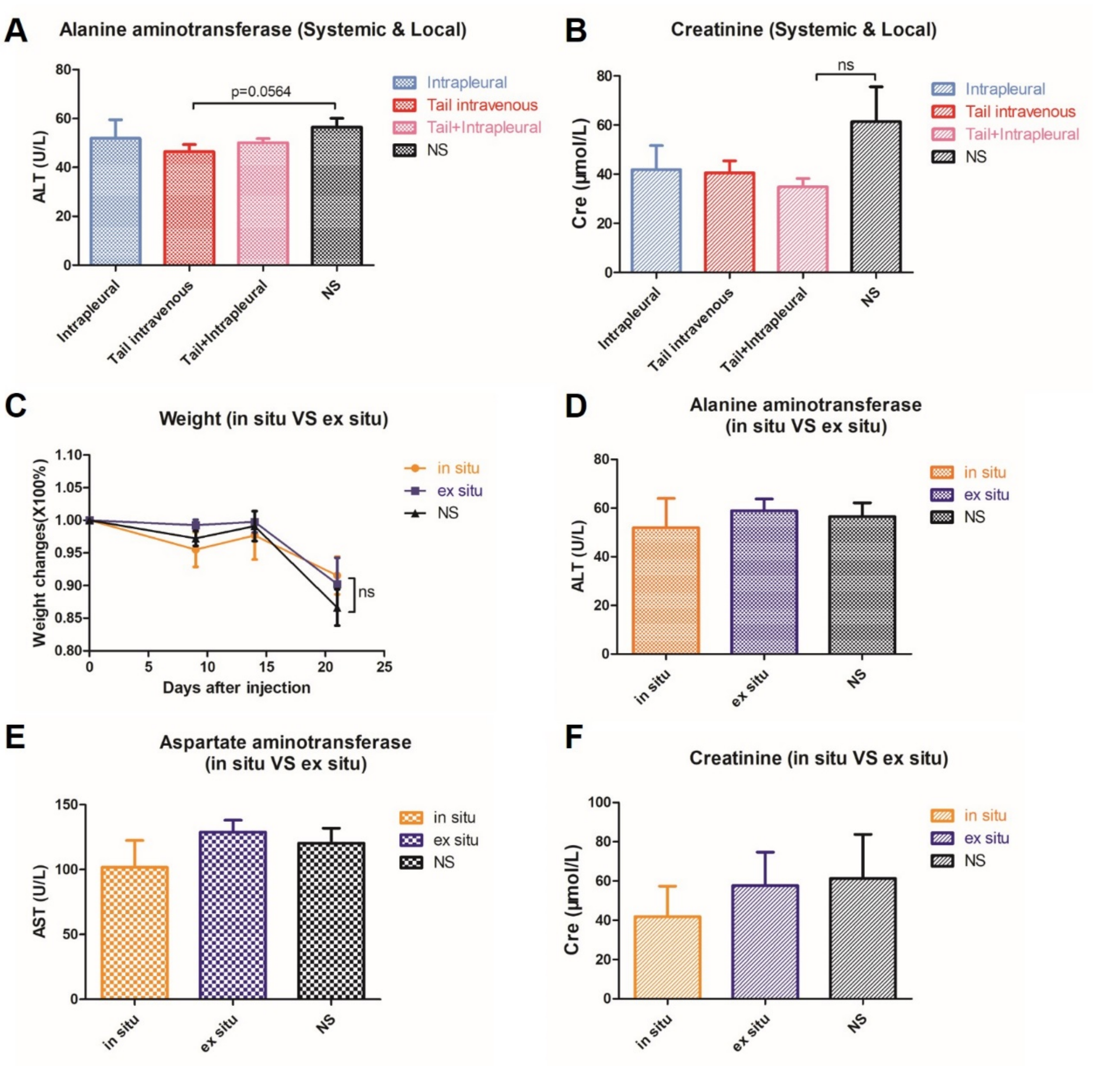

Figure S2. Safety evaluation of intrapleural injection of Anti-PD1 mAb in mice. (A) Blood alanine aminotransferase (ALT) levels of the four groups of mice with different injection methods were shown by the histogram. (B)Serum creatinine (Cre) levels in the above 4 groups were found in the histogram. (C) Comparison of contralateral and in situ, NS group mouse body weight changes after modeling. (D) (E) (F) Histogram of the contralateral administration of mice blood alanine aminotransferase (ALT), blood aspartate aminotransferase (AST) and blood creatinine (Cre) levels. Independent sample T test. * P <0.05; ** P <0.01, *** P <0.005; ns, P> 0.05.

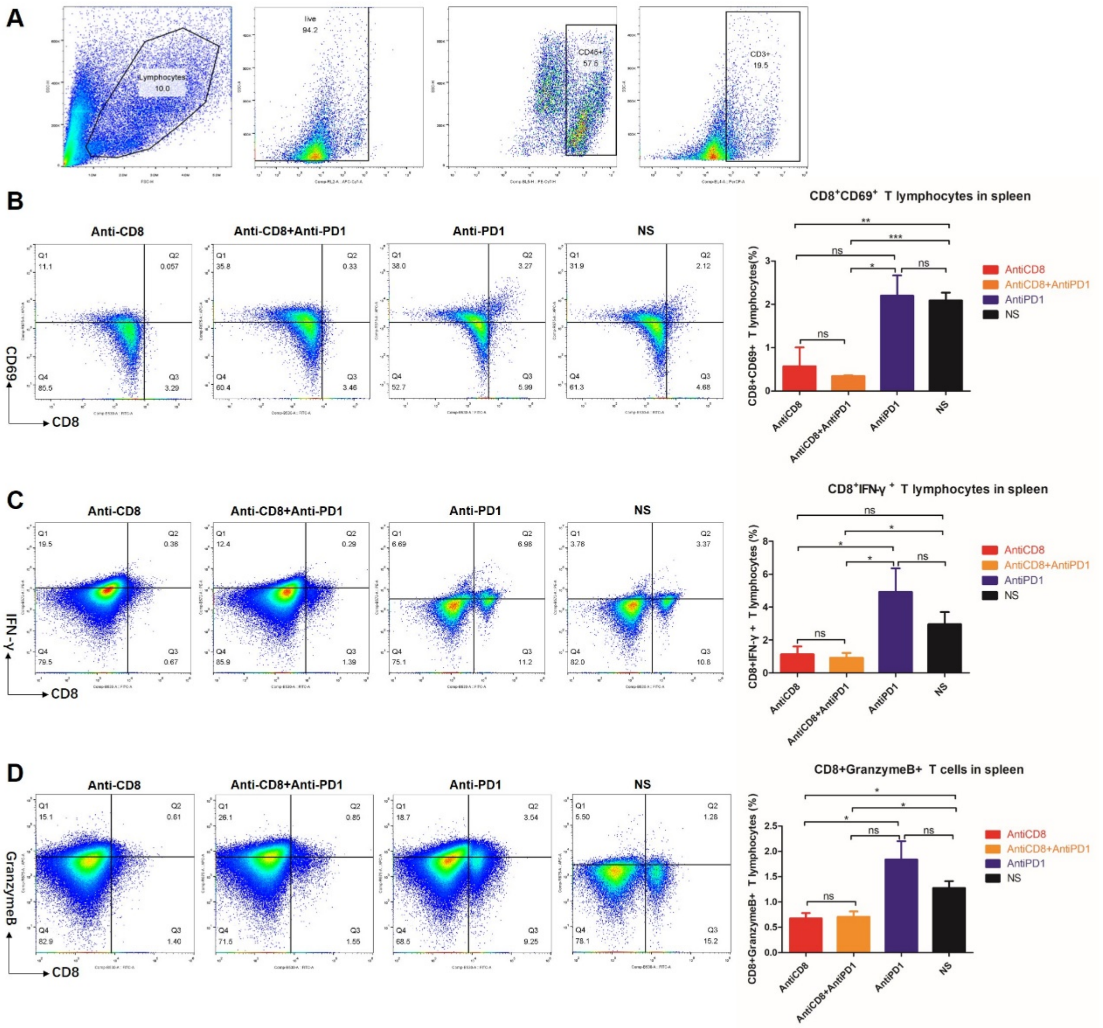

Figure S3. Splenic CTL activity and function were not affected after intrapleural injection of Anti-PD1 mAb. (A) Flow cytometry circled live cells, CD45 + cells, and CD3 + cell populations in sequence, and determined that the analyzed cells were T lymphocytes in local cancer. (B) The percentage of CD8+CD69+ cells in all lymphocytes was compared among 4 groups of local cancers. (C) Histogram comparison of the proportion of CD8+ IFN-γ+ cells in T lymphocytes in spleen of the above 4 groups of mice. (D) The proportion of CD8+ granzyme B+ T cells in the spleen of the above 4 groups could be seen in the histogram. Independent sample T test. * P <0.05; ** P <0.01, *** P <0.005; ns, P> 0.05.

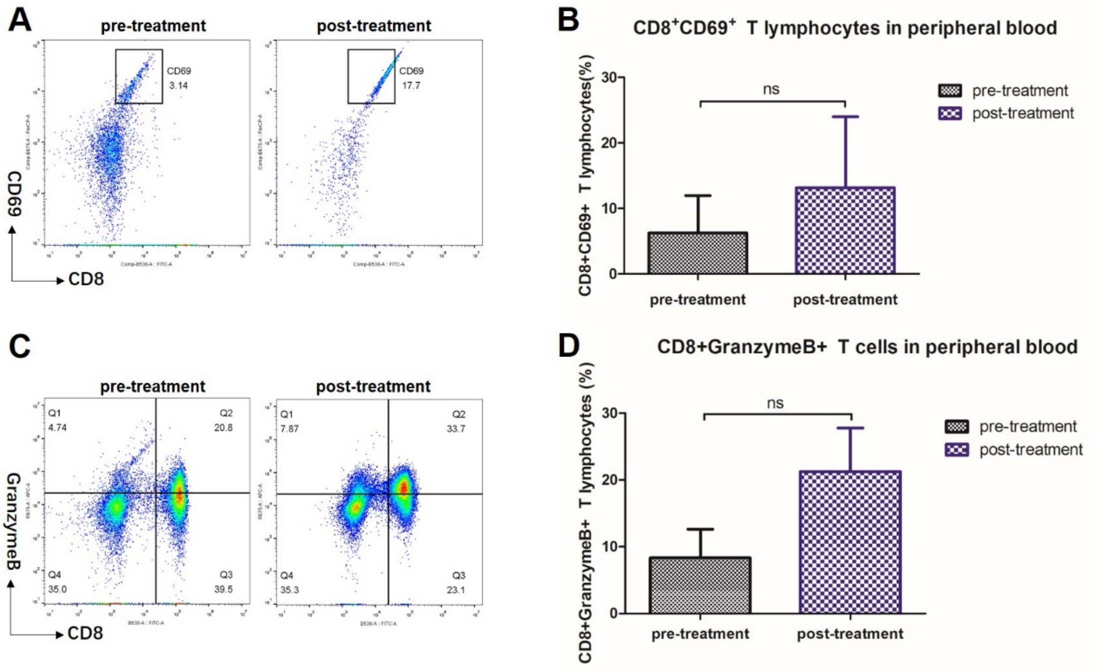

Figure S4. Effect of local Anti-PD1 monoclonal antibody treatment on the activity and function of CTL in peripheral blood of patients. (A) Flow density map showed the CD8+CD69+ cell population in peripheral blood before and after intrathoracic injection of Anti-PD1. (B)Histograms were used to calculate the proportion of CD8+CD69+T cells in peripheral blood of patients. (C)Flow density map showed CD8+ Granzyme B+ cell population in MPE before and after Anti-PD1 treatment. (D)Histogram the proportion of CD8+ Granzyme B+ cells in MPE before and after Anti-PD1 treatment was observed. Independent sample T test. * P <0.05; ** P <0.01, *** P <0.005; ns, P> 0.05.
