# Supplementary material for: Intrapleural Injection of Anti-PD1 Antibody: A Novel Management of Malignant Pleural Effusion
Source: Front Immunol. 2021 Dec 13;12:760683. doi: 10.3389/fimmu.2021.760683 (PMC8711587; doi:10.3389/fimmu.2021.760683)
Supplement: Supplementary file 2 [file Table_1.docx]

Supplementary Table

**Table S1. Primer sequences.**

| β-actin | Forward | 5’-GGCTGTATTCCCCTCCATCG-3’ |
| --- | --- | --- |
|  | Reverse | 5’-CCAGTTGGTAACAATGCCATGT-3’ |
| TNF-α | Forward | 5’-TCCTGGCCAACGGCATGGAT-3’ |
|  | Reverse | 5’-AATCGGCTGACGGTGTGGGT-3’ |
| IL-1β | Forward | 5’-GCAACTGTTCCTGAACTCA-3’ |
|  | Reverse | 5’-CTCGGAGCCTGTAGTGCAG-3’ |
| CCL20 | Forward | 5’-GCCTCTCGTACATACAGACGC-3’ |
|  | Reverse | 5’-CCAGTTCTGCTTTGGATCAGC-3’ |
| CCR6 | Forward | 5’-TGGGCCATGCTCCCTAGAA-3’ |
|  | Reverse | 5’-GGTGAGGACAAAGAGTATGTCTG-3’ |
| Tcf7 | Forward | 5’-AGCTTTCTCCACTCTACGAACA-3’ |
|  | Reverse | 5’-AATCCAGAGAGATCGGGGGTC-3’ |
| Lef1 | Forward | 5’-GCCACCGATGAGATGATCCC-3’ |
|  | Reverse | 5’-TTGATGTCGGCTAAGTCGCC-3’ |
| CXCR5 | Forward | 5’-ATGAACTACCCACTAACCCTGG-3’ |
|  | Reverse | 5’-TGTAGGGGAATCTCCGTGCT-3’ |
| BTLA | Forward | 5’-TGCTTGGGACTCCTCGGTTAT-3’ |
|  | Reverse | 5’-ACACAGATTGTTCCATTGTGCT-3’ |
| FasL | Forward | 5’-TCCGTGAGTTCACCAACCAAA-3’ |
|  | Reverse | 5’-GGGGGTTCCCTGTTAAATGGG-3’ |

Clinical study entry criteria

**Inclusion criteria:** a. the initial occurrence of pleural effusion was confirmed by pathology or cytology to be malignant, and the pleural effusion was moderate or above, which was judged by the clinician to require intervention; b. Pathologically diagnosed as non-small cell lung cancer; c. ECOG score: 0-2; d. Blood routine and biochemical tests all met the normal range, and there was no abnormal function of major organs. There was no recent history of blood transfusion; e. All subjects voluntarily participated in and signed the informed consent to cooperate with the follow-up.

**Exclusion criteria:** a. Patients with contraindications for puncture treatment; b. Patients have contraindications for immunotherapy (including long-term use of hormones, history of radiation pneumonia, etc.); c. Presence of active autoimmune diseases; d. Presence of active infectious diseases (HBV, HCV, HIV, tuberculosis, etc.); e. History of allogeneic organ transplantation and transplantation of somatic hematopoietic stem cells; f. Those who participated in clinical trials of other antitumor drugs within the first 4 weeks; g. Patients who had used PD1/PD-L1 and other immunotherapy drugs.
